# Behavioral measures and self-report of impulsivity in bipolar disorder: no association between Stroop test and Barratt Impulsiveness Scale

**DOI:** 10.1186/s40345-016-0057-1

**Published:** 2016-08-17

**Authors:** Elisa Sophie Strasser, Paula Haffner, Jana Fiebig, Esther Quinlivan, Mazda Adli, Thomas Josef Stamm

**Affiliations:** 1 Dept. of Psychiatry and Psychotherapy, Charité Universitätsmedizin Berlin, Charitéplatz 1, 10117 Berlin, Germany; 2 Fliedner Klinik Berlin, Berlin, Germany

**Keywords:** Bipolar disorder, Impulsivity, Inhibition, Stroop test, Barratt Impulsiveness Scale, Euthymic

## Abstract

**Background:**

Impulsivity as a tendency to act quickly without considering future consequences has been proposed as a dimensional factor in bipolar disorder. It can be measured using behavioral tasks and self-report questionnaires. Previous findings revealed patients to show worse performance on at least one behavioral measure of impulsivity. Additionally, self-reported impulsivity seems to be higher among bipolar patients, both parameters being possibly associated with a more severe course of illness. In this study, our primary aim was to investigate the relationship between these two constructs of impulsivity among bipolar patients.

**Methods:**

A total of 40 euthymic patients with bipolar disorder (21 female, 22 Bipolar I) and 30 healthy controls were recruited for comprehensive neuropsychological assessment. To assess inhibition control as a behavioral measure of impulsivity, the Stroop Color and Word Test (Stroop) was used. Additionally, both groups completed the Barratt Impulsiveness Scale (BIS) as a self-report of impulsivity. To compare the groups’ performance on the Stroop and ratings on the BIS, the non-parametric Mann–Whitney *U* test was used. Within the bipolar group, we additionally examined the possibility of an association between Stroop performance and BIS total scores using Pearson’s Correlation *r*.

**Results:**

Patients and controls differed significantly on the Stroop and BIS, with patients performing worse on the Stroop and scoring higher on the BIS. However, there was no association between the Stroop and BIS within the bipolar group. As an exploratory analysis, a positive correlation between Stroop performance and number of episodes was found. Further, we detected a statistical trend in the direction of poorer Stroop performance among patients treated with polypharmacy.

**Conclusions:**

Both difficulties with behavioral inhibition and self-reported impulsivity were observed to be higher in bipolar patients than controls in the current study. However, within the patient group we did not observe an association between patients’ behavioral performance and self-report. This indicates that the parameters likely constitute distinct, dimensional factors of bipolar disorder. In future research, studies with larger samples should investigate which of the two markers constitutes the better marker for the illness and is more suitable to differentiate the most severe patients.

## Background

The term “bipolar disorder” implicates a disorder with both manic/hypomanic and depressive episodes (APA 1994). Thus, patients seem to experience either one extreme or the other. However, this definition disregards a number of factors that are present throughout all phases of the illness, including euthymia (Levy and Manove 2012). For this reason, establishing a better understanding of such particular dimensional factors present in bipolar disorders is warranted (Henry and Etain 2010). This may be an interesting area of inquiry, as dimensional factors may represent indicators for specific treatment response and thus guide treatment. If subgroups of patients with specific dimensional characteristics were to be identified, it could help investigate possible pathophysiological mechanisms (Henry and Etain 2010).

For instance, impulsivity as one possible dimensional factor in bipolar disorder (Henry and Etain 2010) implicates the tendency to act quickly without considering future consequences (Hamilton et al. 2015). Impulsivity can be measured using behavioral tasks and by self-report questionnaires (Hamilton et al. 2015).

Inhibition control reflects a behavioral manifestation of impulsivity (Newman and Meyer 2014), and constitutes one of the core domains of executive function, which can be divided into response inhibition and interference control (Diamond 2013). Interference control can be measured using the Stroop Color and Word Test (Stroop) (Stroop 1992). Interference control constitutes a gating mechanism, which helps to ignore irrelevant information (Wilson and Kipp 1998) and enhances the ability to suppress stimuli that would ordinarily trigger a competing reaction. Additionally, it activates the ability to suppress distractors which would ordinarily delay the response (Nigg 2000). Dempster (1992, p. 47) emphasized the importance of *“the ability to inhibit or deactivate stored information”* as being *“just as decisive as the quantity and quality of stored information and the availability of activation resources”*.

Another aspect of inhibition involves the ability to control attention, behavior, thoughts, and emotions, as well as the ability to resist internal or external urges or temptations (Diamond 2013). This definition of inhibition is similar to the construct of self-reported impulsiveness applied by Patton et al. (1995) who developed the Barratt impulsivity scale (BIS). The BIS is a 30-item rating scale, where each item is related to one of three second-order facets of impulsivity: These include *attentional impulsiveness* referring to quick cognitive decision-making, *motor impulsiveness* which refers to acting without thinking, and *non*-*planning impulsiveness* which refers to a lack of future planning (Patton et al. 1995).

Both behavioral and self-reported impulsivity implicate important clinical consequences. Inhibition control—or more precisely, interference control, measured by Stroop—may represent a possible endophenotype of bipolar disorders, given that even non-afflicted first-degree relatives of individuals with bipolar disorder seem to show poorer Stroop performance (Arts et al. 2008). Furthermore, an association has been found between decreased interference control and period of time to recovery among first-episode patients (Gruber et al. 2008) as well as between decreased interference control and unemployment among bipolar patients (Ryan et al. 2013). High impulsivity scores measured by the BIS are associated with increases in overall functional impairment (Jimenez et al. 2012), a higher number of episodes at early onset and a higher number of past suicide attempts (Swann et al. 2009), as well as with increases in substance consumption, including alcohol (Nery et al. 2013) and nicotine (Heffner et al. 2012).

Previous findings revealed significant differences between bipolar patients and healthy controls in terms of both the BIS as self-reported impulsivity (Swann et al. 2001, 2003, 2004; Peluso et al. 2007; Kathleen Holmes et al. 2009; Strakowski et al. 2010; Ekinci et al. 2011; Lombardo et al. 2012; Henna et al. 2013; Etain et al. 2013) and the Stroop as behavioral impulsivity (Robinson et al. 2006; Torres et al. 2007; Arts et al. 2008; Kurtz and Gerraty 2009; Bora et al. 2009; Mann-Wrobel et al. 2011). Furthermore, what has been perplexing to date has been the huge variance in performance on the Stroop in a number of meta-analyses, with little explanation of why this may be the case (Robinson et al. 2006; Torres et al. 2007; Arts et al. 2008; Kurtz and Gerraty 2009; Bora et al. 2009; Mann-Wrobel et al. 2011; Hajek et al. 2013). A recent review summarized several studies investigating either behavioral or self-reported impulsivity, which revealed predominantly significant differences in self-reported, but not behavioral tests of impulsivity (Newman and Meyer 2014). However, few studies have examined the link between these two constructs, and it is notable that to date no study has investigated the relationship between the Stroop and the BIS among bipolar patients as its primary research question. If observed, a positive relationship could further support the clinical utility of the BIS as an easily administrated, economical screening tool when assessing bipolar patients. In addition to existing knowledge about the BIS and course of illness, suicidality and substance misuse, it may be possible to gain a more nuanced understanding of behavioral impulsivity’s relationship with these phenomenon. This would have positive implications for clinical practice, insofar as it would aid clinicians in making a brief yet detailed assessment of a patient’s presentation and clinical needs.

The current research aimed to investigate the relationship of self-reported impulsivity (measured by the BIS) and a behavioral measure of inhibition control (Stroop test) in bipolar patients. Initially, we sought to confirm previous findings that bipolar patients show a poorer performance on the Stroop and a higher BIS score compared to healthy controls. Then, as main research question, we sought to examine whether poorer performance on a behavioral test of impulsivity was related to higher self-reported impulsivity in a group of bipolar patients.

Additionally, we wanted to investigate a possible association between impulsivity measures and possible confounders, such as number of episodes, subthreshold depressive symptoms, medical treatment, and years of education in an exploratory analysis.

## Methods

### Participants

A total of 50 bipolar patients (29 female, 27 Bipolar I) and 43 healthy controls were seen for a comprehensive neuropsychological assessment. All patients were recruited from the psychiatric outpatient clinic at the Charité Mitte Campus University Hospital in Berlin based on the following inclusion criteria: diagnosis of bipolar disorder according to the DSM-IV; clinical remission meeting the criteria of euthymia [Hamilton Depression Rating Scale version 21 (HAMD-21) (Hamilton 1960) ≤9 and Young Mania Rating Scale (YMRS) (Young et al. 1978) ≤12] for at least 6 weeks; absence of affective symptoms; medication with a mood stabilizer for at least three months; minimum age of 18 years. A number of strictly euthymic patients were systematically looked for, to achieve a broad range of patients’ composition (i.e., those with a HAMD-21 ≤ 3, based on practice in a recent study regarding subthreshold symptoms in bipolar disorder) (Bonnin et al. 2012). Patients were excluded if they met the criteria of current psychotic symptoms, substance abuse during the last three months, dementia or mild cognitive impairment, or other predominant Axis I disorder within the past six months. Diagnoses using DSM-IV were undertaken by experienced and trained assessors with more than five years of experience with clinical diagnostics. YMRS and HAMD-21 were administered by well-trained assessors.

Healthy controls were recruited by web advertisement and word of mouth and were at least 18 years old. Criteria for exclusion were diagnosis of any current or past Axis1 disorder, assessed by the Mini-International Neuropsychiatric Interview (M.I.N.I.) (Sheehan et al. 1998), and first-degree relatives with an affective disorder or schizophrenia.

From the 50 patients originally recruited, ten were excluded for the following reasons: five emerged to be in a depressive mood state during testing, one emerged to have a mild cognitive impairment, three were not medicated with a mood stabilizer and one was not in a euthymic state for the required minimum six weeks. Of the 43 healthy controls, nine were excluded on the basis of a depressive episode (current or lifetime) or current substance abuse. To avoid the emergence of an age effect on the Stroop task (Comalli et al. 1962), we ensured that participants in both groups were of similar age by systematically removing the four youngest of the 34 healthy controls fulfilling the inclusion criteria.

Patients and controls within this study concurrently participated in two different studies using the same neuropsychological assessment: 34 patients participated in a pilot study investigating the feasibility of metacognitive training for low-functioning bipolar patients (Haffner et al. 2016), and 16 patients participated in a study on cognitive vulnerability in bipolar patients (Quinlivan et al. 2016).

### Assessment

A number of previous studies have used the Stroop as a measure of inhibitory control (Enticott et al. 2006; Kemps and Wilsdon 2010). In the current study, to measure a lack of inhibitory control as a behavioral manifestation of impulsivity [as has been previously indicated (Newman and Meyer 2014)] a German version of the Stroop interference (Bäumler and Stroop 1985) was applied. The outcome variable used was the time needed to complete the test. In the absence of a discrete measure of behavioral impulsivity, the use of a measure of inhibitory control was considered an appropriate alternative. The German version of the Stroop test shows an internal consistency of 0.97 and a retest reliability of 0.93. The facture structure as well as convergent and divergent validity have been confirmed (Bäumler and Stroop 1985). To assess self-reported impulsivity, the BIS-11 questionnaire was used. Regarding validity and reliability of the German version of the BIS-11 scale, the BIS total score showed adequate internal consistencies (Preuss et al. 2008) and findings of a study investigating adolescents ascertained convergent validity and suggested appropriate reliability (Hartmann et al. 2011).

As an interviewer-administered rating scale for the impairment of psychosocial functioning in bipolar disorder, the Functional Assessment Short Test (FAST) (Rosa et al. 2007) was administered with both patients and controls.

To assess general neurocognitive functioning, including executive functions, verbal memory, intelligence and attention, all participants completed a neuropsychological test battery. Executive functions were assessed by a German word fluency task (Regensburger Wortflüssigkeitstest) (Aschenbrenner et al. 2000) and digit span backwards subtest of the German version of the Wechsler memory scale (WMS) (Härting et al. 2000). Verbal memory was measured by the German verbal learning and memory test (Verbaler Lern- und Merkfähigkeitstest, VLMT) (Helmstaedter et al. 2001) and the digit span forward as a subtest of the WMS. The subtest LPS3 of a German intelligence test battery (Leistungsprüfsystem, LPS) (Horn 1983) was used to assess logical thinking as fluid intelligence whereas a multiple choice vocabulary test (Mehrfach Wortschatz Test, MWT-B) (Lehrl 2005) measured crystallized intelligence. Furthermore different aspects of attention and executive function were examined using subtests (alertness and divided attention) of a German computerized test battery (Testbatterie zur Aufmerksamkeitsprüfung, TAP) (Zimmerman and Fimm 2006). A well-trained assessor delivered the comprehensive neuropsychological battery and all participants were tested in similar circumstances concerning place, time, person and instructions.

### Data analysis

As a number of our variables were not normally distributed, use of the non-parametric Mann–Whitney *U* test was indicated. The test was completed to compare patients and healthy controls on a range of variables, including demographics, clinical features and facets of the neuropsychological assessments. Fisher’s exact test was applied on normative variables (e.g., gender). For our confirmatory analyses, the Mann–Whitney *U* test was used to compare patients and healthy controls concerning time needed in the Stroop and scoring in the BIS (both in terms of total and subscale scores). Because of patients and controls differing on the BDI, a regression analysis was conducted to explore, whether the BDI predicts Stroop and, respectively, BIS. A second Mann–Whitney *U* test was then applied to compare the 15 strictly euthymic patients’ (with HAMD-21 ≤ 3) and controls’ Stroop performance and BIS scores. In terms of our primary question, time needed in the Stroop interference and BIS total scores was correlated according to Pearson within the patient group. To investigate possible confounders of the Stroop test probably being the more robust measure, exploratory analyses were conducted. This was completed by correlating time needed in the Stroop interference with six possible confounders available in our dataset, such as subthreshold depressive symptoms (as measured by the HAMD-21), subthreshold manic symptoms (as measured by the YMRS), years of education, duration of illness, number of hospitalizations and the FAST cognitive score. Because of the explorative character of these correlations, we did not perform a type II error correction. Data analyses were conducted using IBM SPSS Statistics Version 22.0.

## Results

Of the 40 patients fulfilling the inclusion criteria, 15 had a HAMD-21 ≤ 3 which indicated that they were strictly euthymic at the time of testing. An overview of the demographics and clinical characteristics is shown in Table 1.

**Table 1:** Demographic and clinical characteristics of bipolar patients (BD) and healthy controls (HC)

Variable	*BD*			*HC*			Statistics
	n	*M/Mdn/n*	Range	n	*M/Mdn/n*	Range	
Gender female	40	21 (52.50 %)		30	18 (60.00 %)		× n.s.
Age (years) *Mdn* (IQR)	40	48.00 (21.00)	23–77	30	38.50 (21.00)	26–63	◊ n.s.
Years of education *Mdn* (IQR)	40	18.00 (5.00)	11–18	30	15.50 (6.00)	11–18	◊ n.s.
Number of episodes *M* (SD)	40	21.78 (15.04)	3–72	–			–
Age at onset *M* (SD)	40	29.10 (11.59)	13–58	–			–
Duration of illness in years *M* (SD)	40	18.00 (10.87)	1–48				
Number of hospitalizations *M* (SD)	40	2.93 (2.94)	0–12				
Prior suicide attempts *M* (SD)	40	0.45 (1.06)	0–5	–			–
Bipolar 1	40	22 (55.00 %)		–			–
Rapid cycling lifetime	40	7 (17.50 %)		–			–
Past psychotic symptoms	39	11 (28 %)					
Number of different psychotropic treatment groups *M* (SD)	40	1.88 (0.79)		–			–
Treatment with lithium	40	19 (47.50 %)		–			–
Treatment with antipsychotics	40	16 (40.00 %)		–			–
Treatment with antiepileptics	40	26 (65.00 %)		–			–
Treatment with antidepressants	40	15 (37.50 %)		–			–
BDI *Mdn* (IQR) (Beck et al. 1961)	39	5.00 (15.00)	0–26	30	2.00 (6.00)	0–10	◊ *z* = −3.08 *p* ≤ .01 *r* = .37
HAMD *M* (SD)	40	5.05 (2, 90)	0–9	–			–
YMRS-D *M* (SD)	40	1.50 (2, 08)	0–9	–			–
FAST general score *Mdn* (IQR)	40	23.00 (16.75)	0–45	30	2.00 (5.00)	0–8	◊ *z* = −6.53 *p* ≤ .001 *r* = .78
FAST cognitive score *Mdn* (IQR)	40	5.00 (5.75)	0–11	30	1.00 (2.00)	0–4	◊ *z* = −5.82 *p* ≤ .001 *r* = .70

*M* mean, *Mdn* median, *SD* standard deviation, *IQR* interquartile range, ◊ Mann–Whitney *U* test, × Fisher’s exact test, n.s. ≥ .05

**Fig. 1:**
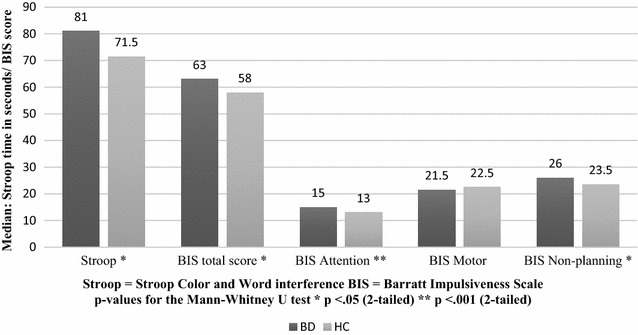
Behavioral and self-reported impulsivity of euthymic patients (n = 40) and healthy controls (n = 30). On the Stroop test, poorer performance is indicated by higher scores (i.e., longer response duration). On the BIS, higher scores also indicated a greater affliction (i.e., more impulsive self-reports). Note: when comparing the subgroup of strictly euthymic patients to healthy controls, only the Stroop and the BIS sub-score *attentional* stay significantly different

First, we observed that patients needed significantly more time to complete the Stroop interference task than healthy controls (*z* = −2.49, *p* = .01, *r* = .30, for all BIS and Stroop scores see Table 2). Patients also scored significantly higher on self-reported impulsivity, as measured by the BIS total score (*z* = −2.08, *p* = .04, *r* = .25). With regard to the three subscales on the BIS, patients scored significantly higher than controls in terms of *attentional impulsiveness* (*z* = −3.67, *p* ≤ .001, *r* = .44) and *non*-*planning impulsiveness* (*z* = −1.98, *p* < .05, *r* = .24). However, for the *motor impulsiveness* subscale no significant differences were observed, see Fig. 1 and Table 2.

**Table 2:** Scores of Stroop and BIS of all euthymic patients (HAMD-21 ≤ 9), the strictly euthymic subgroup of patients (HAMD-21 ≤ 3) and healthy controls

Variable	Euthymic patients *n* = 40		Strictly euthymic patients *n* = 15		Healthy controls *n* = 30	
	*Mdn* (IQR)	*M* (SD)	*Mdn* (IQR)	*M* (SD)	*Mdn* (IQR)	*M* (SD)
Stroop	81.00 (27.50)	82.65 (18.16)	88.00 (31.00)	85.27 (17.38)	71.50 (17.5)	73.53 (16.56)
BIS total	63.00 (16.00)	63.20 (9.71)	63.00 (18.00)	63.33 (9.91)	58.00 (7.75)	58.23 (7.67)
BIS *attention*	15.00 (5.00)	16.10 (3.54)	15.00 (5.00)	15.07 (2.76)	13.00 (3.00)	13.17 (2.51)
BIS *motor*	21.50 (4.75)	21.88 (3.71)	22.00 (5.00)	22.80 (3.78)	22.50 (4.25)	22.03 (3.09)
BIS *non*-*planning*	26.00 (6.75)	25.23 (4.64)	26.00 (8.00)	25.47 (5.22)	23.50 (5.00)	23.03 (4.55)

*M* mean, *Mdn* median, *SD* standard deviation, *IQR* interquartile range

Because of the above-analyzed groups differing on the BDI (a self-rating scale for depressive symptoms, see Table 1), additional analyses were run: A regression analyses showed that concerning the whole sample of patients and controls, the BDI could not significantly explain any variance of the Stroop (*R*^2^ = .00, *p* = .88) whereas it could explain 20.3 % of variance of the BIS (*R*^2^ = .20, *p* < .001). Thus, respecting the BDI as a possible confounder in the comparison between patients and controls, in a second step an additional comparison between the 15 strictly euthymic patients (HAMD-21 ≤ 3) and the 30 healthy controls was conducted. Groups did not differ on the BDI, nor concerning age or, respectively, gender (all *p* values >.05). Now, patients and controls significantly differed only on the Stroop (*z* = −2.25, *p* = .02, *r* = .34), but not on the BIS total score (*z* = −1.68, *p* = .09). Regarding the BIS sub-scores, only the BIS *attentional* remained significantly different between patients and controls (*z* = −2.26, *p* = .02, *r* = .34), whereas there was no statistical difference concerning the sub-scores *non*-*planning* and *motor* (all *p* values >.05).

In regard to our primary research question, data showed no significant positive correlation between patients’ test performance on the Stroop and total BIS scores (*n* = 39 due to the exclusion of one outlier on the Stroop, *r* = −.09, *p* = .60). Similarly, we did not observe any significant correlations when we examined time needed on the Stroop with the respective BIS subscales (*attentional, motor* and *non*-*planning* impulsiveness). Therefore, in the current study’s sample of bipolar patients, self-reported impulsivity was not related to behavioral inhibition performance on the Stroop. The two constructs were positively correlated neither regarding healthy controls (*n* = 29 due to the exclusion of one outlier on the Stroop, *r* = .13, *p* = .49) nor in the whole sample (*n* = 69, due to the exclusion of one outlier on the Stroop, *r* = .06, *p* = .61).

The Stroop showed to possibly constitute a more exact measure which seems more independent of current symptoms than the BIS. Therefore, possible associations between the Stroop and six possible confounders were further explored. We observed a significant correlation between time needed on the Stroop and number of mood episodes (*n* = 39 due to one outlier; *r* = .34; *p* = .03). We also observed a trend in the direction of significance for time needed on the Stroop and number of different psychotropic medication groups (n = 39, *r* = .31; *p* = .06). However, there was no association between Stroop performance and subthreshold depression (as measured by the HAMD-21), subthreshold manic symptoms (as measured by the YMRS), years of education, duration of illness and number of hospitalizations (all *p*’s > .05). Regarding the FAST Cognitive score, there was a positive correlation with Stroop performance (*r* = .339; *p* = .04).

With respect to our descriptive analysis of neurocognitive function, patients performed similar to healthy controls across nearly all domains of the neuropsychological test battery. Thus, there were no significant differences in tests of memory, attention and intelligence. Regarding executive functions, patients showed significantly worse results in the two word fluency tests (i.e., word fluency naming animals: *Mdn* patients = 24.00, *Mdn* controls = 26.50, *z* = −2.21, *p* = .03, *r* = .26; and word fluency S-words: *Mdn* patients = 14.00, *Mdn* controls = 16.50, *z* = −2.36, *p* = .02, *r* = .28). As regards to number of errors in the Stroop interference task, no significant difference was observed between the groups; however, there was a trend in this direction (*M* patients = .97 *Mdn* patients = .00, *M* controls = .27 *Mdn* controls = .00, *z* = −1.91, *p* = .06, *r* = .23).

## Discussion

Aside from exploratory research, this study is the first to comprehensively investigate the relationship between behavioral and self-reported impulsivity, using the Stroop Test and the BIS in a sample of bipolar patients. Patients showed poorer Stroop performance and higher BIS scores than controls, yet our most striking finding was the absence of a positive correlation between Stroop performance and BIS reports within the bipolar group. Moreover, our study revealed promising exploratory findings regarding the relationship of inhibition control and number of episodes and medication.

A notable strength of the current study lies in the range of patients sampled, including a number of strictly euthymic patients and a subgroup of particularly low-functioning patients (see Table 1). Thus, we have accounted for and considered the diversity of bipolar patients and minimized possible important biases when comparing patients and controls on their test performance. For the healthy controls for instance, we ensured an absence of any first-degree relatives with an affective disorder or schizophrenia, given the potential for inhibition and impulsivity as possible endophenotypes. The detailed neurocognitive test battery facilitated us to achieve a broad profile of the sample, supporting the study’s strength.

### Comparing Stroop performance of patients and healthy controls

We found a significant difference between patients’ and controls’ Stroop test performance with a medium effect size. This is in accordance with previous meta-analytical findings (Robinson et al. 2006; Torres et al. 2007; Arts et al. 2008; Kurtz and Gerraty 2009; Bora et al. 2009; Mann-Wrobel et al. 2011), with the exception of one (Hajek et al. 2013). It is notable that even when comparing the group of strictly euthymic patients to healthy controls, the difference remains statistically significant.

### Comparing BIS scores of patients and healthy controls

Equally, in terms of self-reported impulsivity, we were able to confirm previous studies in which bipolar patients showed a higher BIS total score than healthy controls (Swann et al. 2001, 2003, 2004; Peluso et al. 2007; Kathleen Holmes et al. 2009; Strakowski et al. 2010; Ekinci et al. 2011; Lombardo et al. 2012; Henna et al. 2013). A study, investigating a total of 504 healthy controls, measured a BIS total score of *M* = 59.25 (SD = 9.31) (Aichert et al. 2012); a finding similar to that of our healthy controls. Thus, patients in our sample are more impulsive than population norms, pointing towards the consideration of impulsivity as a trait characteristic of bipolar disorder, independent of current illness phase.

It should be noted, however, that there is an association between the BIS and self-report of depressive symptoms, suggesting that the BIS as a self-report might not be suitable as a trait marker. When comparing the group of strictly euthymic patients to the healthy controls, the difference concerning the BIS total score was no more significant (although the strictly euthymic subgroup showing the same BIS total score as the whole bipolar sample). Only the BIS sub-score *attentional* stayed significantly different between patients and healthy controls. This might imply that the BIS attentional could be the more exact measure. This would be in accordance with a previous study on the early diagnosis of bipolar disorder, where the BIS sub-score *attentional,* but not the total score, showed to be a good marker predicting onset of (hypo)mania in subjects at risk (Ng et al. 2016).

Two studies reported an absence of differences (Christodoulou et al. 2006; Lewis et al. 2009) between patients and healthy controls, indicating that this research question warrants thorough inquiry.

### The relationship between behavioral and self-reported impulsivity in bipolar disorder

In our findings, there was no relationship between Stroop interference and the BIS; neither in terms of the total or subscale scores. Based on how items are constructed in relation to concentration and distraction on the BIS *attention* subscale, it would in fact have been expected that this subscale would be the most likely to correlate with Stroop performance. Our findings are in accordance with exploratory results of one study (Powers et al. 2013), which similar to us observed a lack of correlation between Stroop performance (amongst seven other neurocognitive test parameters) and the BIS. Thus, the present study can confidently confirm this exploratory finding and support a recent review which proposed that self-report and behavioral measures of impulsivity might indeed reflect distinct theoretical constructs (Newman and Meyer 2014).

In terms of other populations—both general and clinical—poorer performance in the Stroop interference has been found to be associated with higher impulsivity. Correlations with the Stroop have been observed within a group of healthy subjects when tested using the BIS (Enticott et al. 2006), as well as with other clinical groups where problems with impulsivity are noteworthy; for example, for patients with borderline personality disorder (Bader and 2010) and bulimia nervosa (Kemps and Wilsdon 2010). On closer examination, however, in the borderline subgroup (Bader and 2010) there were multiple inventories of impulsivity correlated with several tests of impulsivity, and no Type II error correction for multiple tests was applied. Further, in the small study which sampled patients with bulimia (*n* = 13), after BIS was entered as a covariate, a significant difference between patients and controls on Stroop performance was no longer observed (Kemps and Wilsdon 2010). Due to the lack of robustness of these findings, it is only possible to conclude that a relationship between BIS and Stroop performance is feasible. In the study with healthy subjects (Enticott et al. 2006), a spatial Stroop was implemented as a reading-independent test, and participants were not older than 51 (unlike in our study, where participants ranged from 23 to 77 years of age). This study correlated four different behavioral paradigms of impulsivity with the BIS and its subscales, revealing that only the Stroop task correlated significantly. It is possible that a relationship between the Stroop and BIS may have been more easily detected in a younger sample, given that age may influence Stroop performance (Comalli et al. 1962). Other studies using a range of different populations did not observe a relationship between the Stroop task and BIS at all (Enticott et al. 2008; Aichert et al. 2012). Interestingly, one study investigated four measures of prepotent response inhibition, including the Stroop, and the BIS in a sample of 504 healthy individuals. While Stroop did not correlate with BIS, a latent variable analysis revealed all four measures of response inhibition to be underpinned by the same construct, where the BIS explained 12 % of the variance (Aichert et al. 2012). In light of these mostly exploratory findings, the current results seem to contribute to a controversial database, where overall there has been, at best, a small relationship between behavioral and self-reported impulsivity when using these particular measures.

Studies using tests other than the Stroop to investigate the relationship between self-reported impulsivity and inhibitory control as a measure of behavioral impulsivity within bipolar patients have partially found evidence for a positive correlation. However, these studies were mostly exploratory. For example, Cheema et al. (2015) found higher BIS scores to be associated with slower reaction times in an emotional Go/No-go test, interpreted by the authors as a possible compensatory cognitive strategy to manage increased impulsivity. However, this correlation was one of many tests run without the use of an error correction, again indicating the possibility for Type II error. Beyond that, the consideration of multiple other test findings are warranted. For instance, higher attentional BIS scores have been associated with a lower response inhibition in the Hayling Sentence Completion test (Christodoulou et al. 2006). BIS *motor score* has been correlated with more impulsive behavior in the Balloon Analogue Risk Task (Kathleen Holmes et al. 2009). However, BIS impulsivity has not been found to be related to decreased inhibition in the Stop Signal Task (Heffner et al. 2012). The discrepancies in findings of theoretically similar constructs render it difficult to make meaningful conclusions regarding the nature of impulsivity. It is notable, however, that these differences may be attributable to a lack of consistency in the methods and measures used; for example, the various procedures of the behavioral tests of impulsivity which were not always shown to inter-correlate (Enticott et al. 2006).

### Influence of possible confounders on Stroop performance

The present study revealed an association between Stroop performance and total number of episodes; strengthening previous findings indicating that number of affective episodes is negatively associated with executive functions (El-Badri et al. 2001). Equally, we observed a trend in a positive correlation between Stroop performance and number of psychotropic medication groups. The influence of medication on cognitive performance has been reported controversially to date. Goswami et al. (2009), for example, did not find any influence of medical treatment on any type of cognitive performance, whereas Bora et al. (2009) reported an association between medication and the magnitude of impairment on psychomotor speed. Considering the Stroop test as a speed-dependent test could explain poorer performance among patients treated with a range of substances. In terms of depressive symptoms, subthreshold symptoms did not influence Stroop performance in our findings, which confirms previous results (Bora et al. 2009).

### General neuropsychological test performance

Compared to healthy controls, in our study euthymic bipolar patients showed a similar test performance across all cognitive domains with the exception of executive function. This is in contrast with previous meta-analyses stating that even in euthymia, bipolar patients show cognitive impairment in nearly all domains (Robinson et al. 2006; Torres et al. 2007; Arts et al. 2008; Kurtz and Gerraty 2009; Mann-Wrobel et al. 2011; Porter et al. 2015). In one study investigating cognitive subgroups, 41.4 % of the bipolar patients did not show any cognitive deficits (Volkert et al. 2015). Again here our study seems to contribute to somewhat of a controversial empirical database. In terms of executive functions, we reported significant differences in word fluency. This is in accordance with several meta-analyses to date, which have all estimated executive functions to be particularly limited in this population (Robinson et al. 2006; Torres et al. 2007; Arts et al. 2008; Kurtz and Gerraty 2009; Bora et al. 2009).

### Limitations

The results of this study are limited by its rather small sample size, though this can be partly counterbalanced by the huge range in our sample composition. Another notable point to consider is that participants were recruited through a university hospital, indicating a possible selection bias. It is possible that patients attending a specialist bipolar clinic received a more frequent, expert careplan than other typical bipolar patients attending community-based services. Beyond that, nearly all of the patients are treated by multiple different medications, which could influence their Stroop performance. Therefore, the statistically significant difference between patients and healthy controls on the Stroop might partly be due to patients’ treatment with polypharmacy. Finally, it should be noted that the broad age range in our sample may have affected a potential correlation between Stroop and BIS (Comalli et al. 1962).

## Conclusions

In our study, both behavioral and self-reported impulsivities were increased within our patient group as compared to controls; however, we did not find a correlation between these two constructs. Thus, our study highlights the importance of considering these aspects of impulsivity as two independent dimensional factors in bipolar disorder, which probably both influence the course of illness and functional outcome in respective ways. Our findings suggest the possible usefulness of specific cognitive trainings for bipolar patients, with a focus on executive functions. Additionally, our findings indicate that it is particularly important to identify and prescribe a pharmacotherapy that does not aggravate cognitive functioning in cases where performance is already compromised, or in cases of an advanced course of illness, to ensure lack of disruption in patients’ quality of life.

In future research, we recommend that studies with a longitudinal design investigate Stroop and BIS on a large sample of bipolar patients. Thus, one could investigate which of the two markers constitutes a better marker for the illness and may, therefore, be more suitable for differentiating the most severe patients (e.g., those with substance misuse, more suicide attempts and a more severe course of illness). Beyond that, a study examining subjects at risk for bipolar disorder who are not medicated yet could further investigate the relevance of interference control as a marker for bipolar disorder.

## Abbreviations

BDI: Beck Depression Inventory
BIS: Barratt Impulsiveness Scale
FAST: Functional Assessment Short Test
HAMD-21: Hamilton Depression Rating Scale (version 21)
M.I.N.I.: Mini-International Neuropsychiatric Interview
Stroop: Stroop Color and Word Test
YMRS: Young Mania Rating Scale
